# Retraction Note: An effective PO-RSNN and FZCIS based diabetes prediction and stroke analysis in the metaverse environment

**DOI:** 10.1038/s41598-026-55044-4

**Published:** 2026-06-03

**Authors:** M. Karpagam, S. Sarumathi, A. Maheshwari, K. Vijayalakshmi, K. Jagadeesh, V. Bereznychenko, R. Narayanamoorthi

**Affiliations:** 1 Department of Computational Intelligence, Faculty of Engineering and Technology, SRM Institute of Science and Technology, SRM Nagar, Tamil Nadu, Kattankulathur, Chennai, 603203 India; 2 Department of Artificial Intelligence and Data Science, K.S.Rangasamy College of Technology, Tamil Nadu, Tiruchengode, 637215 India; 3 Department of Computer Science and Engineering, School of Computing, Vel Tech Rangarajan Dr. Sagunthala R&D Institute of Science and Technology, Avadi, Chennai, 600062 India; 4 Department of Theoretical Electrical Engineering and Diagnostics of Electrical Equipment, Institute of Electrodynamics, National Academy of Sciences of Ukraine, Beresteyskiy Avenue, 56, Kyiv, 03057 Ukraine; 5 Department of Electrical and Electronics Engineering, SRM Institute of Science and Technology, Kattankulathur, Chennai, Tamilnadu, 603 203 India

Retraction of: *Scientific Reports* 10.1038/s41598-025-96541-2, published online 04 April 2025

The Editors have retracted this Article.

After publication, concerns were raised regarding the datasets used in this study [1]. The Authors relied on the publicly available datasets, the Stroke Prediction Dataset and the Diabetes Prediction Dataset, whose provenance and validity cannot be confirmed. As these datasets were used for the training and validation of the proposed model in this Article, the Editors no longer have confidence in the reliability of the results and conclusions of this Article.

M. Karpagam disagree with this retraction. S. Sarumathi, A. Maheshwari, K. Vijayalakshmi, K. Jagadeesh, V. Bereznychenko, and R. Narayanamoorthi did not respond to correspondence from the Editors about this retraction.
